# Immunological and Biochemical Characterization of Coxsackie Virus A16 Viral Particles

**DOI:** 10.1371/journal.pone.0049973

**Published:** 2012-11-30

**Authors:** Pele Chong, Meng-Shin Guo, Fion Hsiao-Yu Lin, Kuang-Nan Hsiao, Shu-Yang Weng, Ai-Hsiang Chou, Jen-Ren Wang, Shih-Yang Hsieh, Ih-Jen Su, Chia-Chyi Liu

**Affiliations:** 1 Vaccine R&D Center, National Institute of Infectious Diseases and Vaccinology, National Health Research Institutes, Zhunan Town, Miaoli County, Taiwan; 2 Graduate Institute of Immunology, China Medical University, Taichung, Taiwan; 3 Department of Medical Laboratory Science and Biotechnology, College of Medicine National Cheng Kung University, Tainan, Taiwan; National University of Singapore, Singapore

## Abstract

**Background:**

Coxsackie virus A16 (CVA16) infections have become a serious public health problem in the Asia-Pacific region. It manifests most often in childhood exanthema, commonly known as hand-foot-and-mouth disease (HFMD). There are currently no vaccine or effective medical treatments available.

**Principal Finding:**

In this study, we describe the production, purification and characterization of CVA16 virus produced from Vero cells grown on 5 g/L Cytodex 1 microcarrier beads in a five-liter serum-free bioreactor system. The viral titer was found to be >10^6^ the tissue culture's infectious dose (TCID_50_) per mL within 7 days post-infection when a multiplicity of infection (MOI) of 10^−5^ was used for initial infection. Two CVA16 virus fractions were separated and detected when the harvested CVA16 viral concentrate was purified by a sucrose gradient zonal ultracentrifugation. The viral particles detected in the 24–28% sucrose fractions had low viral infectivity and RNA content. The viral particles obtained from 35–38% sucrose fractions were found to have high viral infectivity and RNA content, and composed of four viral proteins (VP1, VP2, VP3 and VP4), as shown by SDS-PAGE analyses. These two virus fractions were formalin-inactivated and only the infectious particle fraction was found to be capable of inducing CVA16-specific neutralizing antibody responses in both mouse and rabbit immunogenicity studies. But these antisera failed to neutralize enterovirus 71. In addition, rabbit antisera did not react with any peptides derived from CVA16 capsid proteins. Mouse antisera recognized a single linear immunodominant epitope of VP3 corresponding to residues 176–190.

**Conclusion:**

These results provide important information for cell-based CVA16 vaccine development. To eliminate HFMD, a bivalent EV71/CVA16 vaccine formulation is necessary.

## Introduction

Coxsackie virus A16 (CVA16), similar to polio virus, is a non-enveloped, single positive-stranded RNA virus of the family *Picornaviridae* with a genome size of approximately 7.4 kb. CVA16 consists of 60 copies of VP1, VP2, VP3 and VP4 capsid proteins that form a pentameric icosahedral particle. Viral infections by CVA16 and enterovirus 71 (EV71) can cause hand-foot-and-mouth disease (HFMD) and herpangina in young children [1], [2]. As such, it has become a serious public health problem in the Asia-Pacific region [1]–[8]. Although the host receptors for EV71 and CVA16 have been identified [9], [10], there is no effective antiviral agent to combat both EV71 and CVA16 infections. Since EV71 infections can lead to severe diseases such as poliomyelitis-like paralysis and fatal encephalitis in neonates [1]–[8], it has attracted greater vaccine research and development [1]–[2]. Experimental vaccines that have been tested in animal models include a live-attenuated virus [8]–[13], formalin-inactivated virions [14], [15], baculovirus expressed virus-like particles [16], VP1 recombinant protein [17], [18], a VP1 DNA vaccine [19], a VP1 peptide-based vaccine [20], [21], bacterial or viral vectors expressing VP1 [17], [22], and a Vero cell-adapted live attenuated virus [13]. Among all these vaccine candidates, formalin-inactivated EV71 (FI-EV71) has been found to be the most potent and is currently being tested in human clinical trials [17], [18], [23], [24]. Mao *et al.* [25] and our unpublished results have shown that animal anti-FI-EV71 sera had low or no cross-neutralization effect against CVA16. Therefore, a bi-valent vaccine containing antigens from both viruses should be developed to prevent HFMD. In the present study, we describe the up-stream bioprocess, down-stream purification, biophysical and immunological characterization of CVA16 viral particles that were produced from Vero cells grown in a serum-free microcarrier bioreactor system.

## Materials and Methods

### Ethics Statement

All experiments were conducted in accordance with the guidelines of the Laboratory Animal Center of NHRI. Animal use protocols have been reviewed and approved by the NHRI Institutional Animal Care and Use Committee (Approved protocol No. NHRI-IACUC-098033-A).

### Cells, culture medium and virus

African green monkey kidney (Vero) cells were kindly provided by the Taiwan Centers of Disease Control (Taiwan CDC), which purchased the cell line from the American Type Culture Collection (ATCC, Manassas, VA USA). In general, Vero cells were grown in serum-free VP-SFM medium (Gibco/Life Technologies, Taipei, Taiwan), and cells were passaged twice weekly in T75 T-flasks. The N5079 strain (CVA16 virus clinical isolate) was obtained from the National Cheng-Kung University (Tainan, Taiwan). The genomic sequences of CVA16 5079 was reported in GenBank: AF177911.1 Tainan/5079/98. CVA16 (N5079) virus stocks were collected from the supernatants of infected Vero cells at three days post-infection (DPI). Furthermore, master virus seed banks were selected from high and fast growth clone (5079N/R_P2) that was established after 2 passages. The titers of the master virus seed stocks were determined by a plaque assay, and the stocks were stored at −80°C. To study the genetic stability of clone 5079N/R_P2, CVA16 virus RNA was extracted by a commercial kit produced by Geneaid (Taoyuan, Taiwan). The extracted viral RNA was amplified using one-step RT-PCR (Promega, Madison, WI USA). Oligonucleotide primer sequences used in this study are available upon request. The amplified DNA was sequenced using an ABI 3730 XL DNA Analyzer (Applied Biosystem Inc., Foster City, CA USA). Nucleotide sequences of VP1 and amino acid sequences of all four structural viral proteins reported in this study are available upon request.

### Virus cultivation using a bioreactor system

CVA16 virus (5079N/R_P2) was cultivated using serum-free VP-SFM medium in a 5 liter bioreactor (NBS, US) based on the microcarrier cell culture bioprocess previously reported [15], [26], [27]. Bioreactor culture medium containing 5 g/L of Cytodex 1 was initially inoculated with 1×10^5^ cells per mL. Cell density reached 2 to 2.5×10^6^ cells per mL after six days of cultivation. The Vero cells were infected with CVA16 at a MOI of 10^−5^. CVA16 virus was harvested and collected from the microcarrier culture supernatants at either 7 or 8 days post-infection (DPI).

### Purification of CVA16 virus particles using sucrose gradient ultracentrifugation

The CVA16 virus culture supernatant was harvested from the bioreactor culture. The cell debris was removed by passage through a 0.65 µm filter (Sartorius, Germany), and the supernatant was concentrated 20-fold with a 100K TFF capsule (Pall). The crude CVA16 concentrate (∼200 mL from each 5 L run) was loaded onto a 10–50% continuous sucrose gradient and centrifuged at 32,000 rpm for three hours using a zonal rotor in a Hitachi CP80 ultracentrifuge. The fractions (50 mL per fraction) at 20 to 40% sucrose were collected and individually dialyzed against three exchanges of 1 L PBS at pH 7.4 (Gibco/Life Technologies, Taipei, Taiwan), then stored at 4°C. The infectivity of the purified virus fraction was assessed by a tissue culture's infectious dose (TCID_50_) assay. The fractions were also subjected to SDS-PAGE and Western blot analyses. The fractions identified to contain CVA16 virus were pooled and concentrated by diafiltration using an Amicon 100K tube (Millipore, Belerica, MA USA) and centrifuged at 3,000× g, then stored at 4°C. The total protein concentration of the purified virus fractions was determined by a BCA protein assay. Half of the purified CVA16 virus fractions (15 mL) was stored at −80°C in 0.5 mL aliquots; the other half was inactivated by 1/4000 (v/v) formalin at 37°C for 3 days and stored at 4°C.

### Determination of viral titer

Viral titers were determined using the TCID_50_ median endpoint. Serially-diluted virus samples (from 10^−1^ to 10^−8^) were added to Vero cells grown in 96-well plates, and 10 replicate samples were used for each dilution. The 96-well plates were incubated for six days at 37°C, and TCID_50_ values were measured by counting cytopathic effects (CPE) on infected Vero cells. The TCID_50_ values were calculated using the Reed-Muench method [28].

### SDS-PAGE analysis and Western blotting

SDS-PAGE analysis of purified CVA16 virus fractions was performed in a NuPAGE 4–12% Bis-Tris Gel (Invitrogen, CA USA) according to the protocol suggested by the manufacturer. For immunoblotting, CVA16 viral proteins were directly electrotransferred onto the PVDF membrane and probed with either MAB979 (Millipore, Belerica, MA USA), mouse anti-CVA16 sera, or rabbit anti-CVA16 sera according to procedures previously reported [27].

### Animal immunogenicity studies

Different amounts of inactivated CVA16 particles (5 or 25 µg) were adsorbed on 2 mL aluminum phosphate (3 mg of aluminum) at room temperature for 3 hours before immunization. A group of 6 female BALB/c mice (6–8 weeks old) and a group of 2 rabbits were immunized intramuscularly (i.m.) with either 0.2 mL (mouse) or 0.5 mL (rabbit) of the alum-absorbed inactivated CVA16 particles and were boosted twice with the same dose at two-week intervals after priming. The immunized mice and rabbits were bled one week after the final boost, and the serum was collected and used to analyze virus neutralization.

### Virus neutralizing assay

Serum samples were collected from immunized mice and inactivated at 56°C for 30 minutes. Each serum sample was added to a microtube and serially diluted two-fold with fresh cell culture medium; 400 µL of a 200-TCID_50_ virus suspension was then added to each tube containing 400 µL of serially diluted serum. After incubation at 4°C for 18–24 hours, 100 µL of serially diluted samples were added to the 96-well plates containing Vero cells. The cultures in the 96-well plates were incubated for 7 days at 37°C, and TCID_50_ values were measured by counting CPE in infected cells. The 50% neutralization inhibition dose (ID_50_) was calculated as the reciprocal of the serum dilution that yielded a 50% reduction in the viral titer using the Reed-Muench method.

### Characterization of CVA16 viral particles by transmission electron microscopy (TEM)

Inactivated CVA16 particles were deposited on a Formvar-coated and carbon-vaporized 200-mesh copper grid. The sample was kept on the copper grid for 15 minutes at room temperature, and excess sample was then removed using filter paper. After washing twice with ddH_2_O, the copper grid was stained with 2% phosphotungstic acid solution for 2 minutes, which was then removed using filter paper. The stained grid was dried for 3 days and observed under a Hitachi H-7650 electron microscope.

### CVA16 viral RNA detection by real-time PCR

Viral RNA was isolated from 250 µL of purified CVA16 particles (40 µg total proteins by BCA analysis) using Trizol LS reagent (Invitrogen) according to the manufacturer's instructions. After precipitation, the RNA pellet was dissolved in RNA-Safeguard reagent solution (GeneMark, Taiwan). CVA16 viral RNA was assessed by quantitative PCR analysis using the LightCycler® 480 Real-Time PCR system according to procedures previously reported [27]. Briefly, the CVA16 specific primer pairs were selected from VP2 region, CVA16F: 5′- TCATCCTCCCTACGCCACTA -3′ (1384–1403 bp) and CVA16R: 5′- TAAGGATGCGTCAGAACTGC -3′ (1424–1443 bp), and set No. 64 CAGCCTGG (Cat. No. 004688635001) from Roche Universal Probe Library Assay Design Center (Roche, Switzerland) was selected as probe. The real-time PCR was evaluated with the FastStart Universal Rrobe Master (Rox) (Roche, Germany). The data were analyzed using the LightCycler Software.

### Peptide-ELISA

One hundred and fifty-three overlapping synthetic peptides were synthesized using Fmoc chemistry by the in-house peptide synthesis facility according to the sequence of VP1, VP2, VP3 and VP4 capsid proteins of CVA16. Each peptide contained 15 amino acids, 10 residues of which overlapped with the adjacent peptides. The reactivity of the antibody to each synthetic peptide was analyzed by an enzyme-linked immunosorbent assay (ELISA) according to the protocol previously reported by Panezutti *et al.* [29].

## Results

### Selection and characterizations of CVA16 virus vaccine strain

After reviewing the historical background and medical information, 3 clinical isolates (91N1679, 96N3050, N5079) were obtained from the National Cheng Kung University (Taiwan) and screened for potential as CVA16 vaccine strains. These virus seeds were first adapted to replicate in Vero cells grown in T-flask cell culture containing Medium-199 plus 5% fetal bovine serum. Based on TCID_50_ assay, the virus titer of these viruses can reach >10^6^ by the fifth day of post-infection. One clinical isolate (N5079) that grew consistently to 5×10^6^ virus titer in the T-flask was selected as the vaccine strain (data not shown). To be compliant with Good Manufacturing Practice (GMP) guidelines and screening for the fast growth and high yield CVA16 virus clone, N5079 clinical isolate was first adapted to grow in Vero cell culture containing serum-free (SF) medium VP-SFM and then plaque purified. High-growth virus clones (5079N/R_P2) were selected and prepared for CVA16 virus seed stocks for further evaluation, including genetic characterization and pilot production in bioreactor cell cultures.

### Genetic characterization of 5079N/R_P2 CVA16

Nucleotide sequences of two selected clones from 5079N/R_P2 were analyzed and the gene sequences were found to be 100% match. To be used as a vaccine strain, the genetic stability of 5079N/R_P2 in passage was analyzed based on nucleotide sequences obtained from the master (2 passages) virus seed, working seed banks and the virus harvested from the production lots. The genomic sequences of these two clones were found to be identical to those found in the original sequence data from Professor Jen-Ren Wang of the National Cheng Kung University (Taiwan). Therefore, these results indicate that the 5079N/R_P2 vaccine strain has strong genetic stability in passage.

### Optimization of CVA16 viral yield

Some cell-based inactivated vaccines such as polio, influenza and hepatitis A are commercial available, but there is little information available on their manufacturing processes and culturing systems. Because of intellectual property rights and proprietary technologies used in these vaccine products, there is limited information on virus and product yield influenced by the composition of culture medium and bioreactor systems. For optimal growth of mammalian cells, serum-containing (SC) medium is necessary as a source of nutrients, hormones and growth factors. These serum factors facilitate the attachment and spreading of cells, and also provide protection against mechanical damage and shear forces. Besides these advantages, SC medium may contain unwanted contaminants that could cause safety concerns. To evaluate the efficiency of CVA16 production in Vero cell grown in either the SC medium or SF medium, four different multiplicities of infection (MOI): 0.01, 0.001, 0.0001 and 0.00001 were first tested and compared for virus growth profile in the microcarrier 1 liter bioreactor. The highest virus titer reached 1 to 5×10^6^ TCID_50_/mL by 6 DPI for all MOI tested in both media (data not shown). Since SC medium did not provide significant advantages over SF medium and to eliminate any potential serum protein contaminations, SF medium was selected as the growth medium for CVA16 vaccine production. To test whether temperature could influence virus growth and yield, CVA16 was grown at various temperatures from 32 to 37°C. The virus growth profile at 32°C was slower, but no significant difference in final virus yield was observed at 6 DPI (data no shown). Thus, the 10^−5^ MOI and 37°C were selected and used in all later experiments.

### Production of CVA16 using Vero cell grown in a serum-free medium microcarrier bioreactor

**Figure 1:**
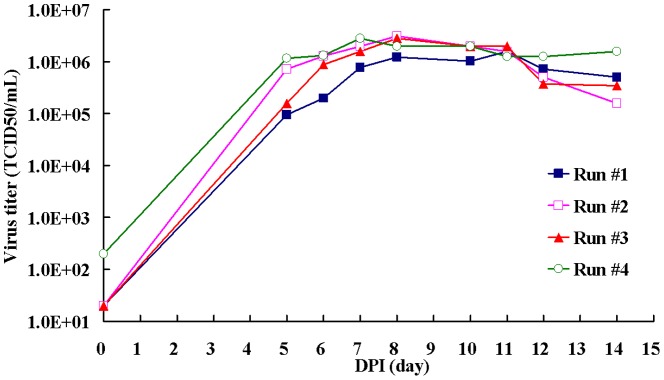
The up-stream process for CVA16 virus production. The consistency of 3 lots of CVA16 virus produced from Vero cells grown in VP-SFM medium (run #1 to 4) in a 5 L microcarrier bioreactor system was monitored. Virus titer was detected every day by TCID_50_ for 14 days.

To establish cell-based CVA16 vaccine production, the serum-free medium microcarrier culture process was used [26], [27]. We found that the viral titers determined by TCID_50_ assay could reach 2×10^6^ TCID_50_/mL at 7 DPI when Vero cells were grown on 5 g/L Cytodex 1 microcarrier bioreactor and infected by CVA16 at 10^−5^ MOI. There was no increase in viral titers at 8 or 9 DPI (Figure 1). This viral titer is 3 to 5-folds lower than those found in the EV71 study [27]. In either 1 or 5 L of bioreactor, the kinetics of CVA16 viral growth based on the viral titer was found to be 2 days slower when compared to EV71 kinetics [27]. When the culture supernatants were harvested at either 7 or 8 DPI, a combination of 0.65 µm filter and the 100K TFF capsule was found to be very effective in removing cell debris and host cell proteins for downstream virus purification (data not shown).

### Purification of CVA16 virus particles using sucrose gradient ultracentrifugation

**Figure 2:**
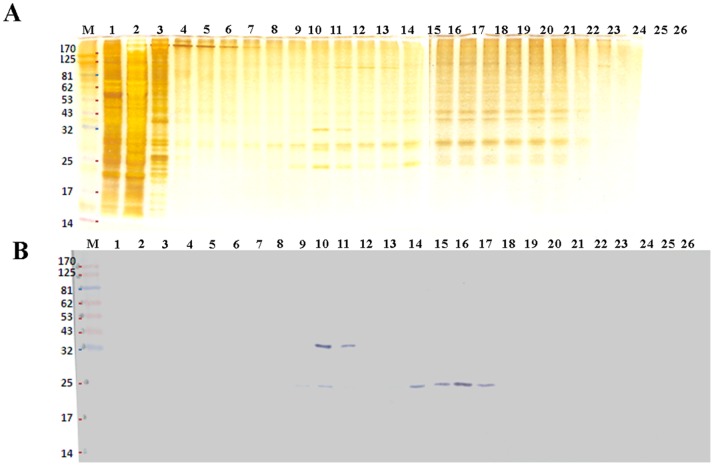
CVA16 purification using sucrose gradient zonal ultracentrifugation. The concentrated CVA16 harvest stock was separated into 25 fractions. (**A**) The viral antigens of each fraction were analyzed by SDS-PAGE and then sliver-stained. (**B**) EV71 antigens were detected by Western blot using MAb979.

A continuous sucrose gradient zonal ultracentrifugation was used to purify CVA16 virus particles from harvest concentrate as previously described [27]. The viral titer and protein concentration of each fraction (50 mL) were determined by TCID_50_ and BCA assays respectively. The highest infectious viral titers were found in fraction 16 containing 36% sucrose, with viral titers reaching 2×10^6^ TCID_50_/mL. Viral titers in the range of 10^5^ to 10^6^ TCID_50_/mL were also detected in fractions 14, 15 and 17. The viral proteins in each fraction were separated by SDS-PAGE (Fig. 2A), and CVA16 viral specific antigens were detected by Western blot using a VP2-specific monoclonal antibody MAb 979 to verify the presence of CVA16 virus particles. CVA16 antigens were found to be separated into two regions of the sucrose gradient (Fig. 2B). The first region containing CVA16 viral antigens was in fractions 10 and 11, which contained 26–28% sucrose and had either low or no infectivity as determined by TCID_50_ assay (data not shown). Based on biochemical, viral and immunological properties, these virus particles were defined as pseudo/defective viral particles or P-particles. The P-particles have: (1) a VP0 protein band (36 kDa) observed in the SDS-PAGE (fractions 10 & 11 of Figure 2A) and they settled at 26–28% sucrose gradient as their biochemical properties; (2) low or no infectivity in the TCID50 assay as the viral property; and (3) VP0 & VP2 protein bands in the Western blot analysis (fractions 10 and 11 in Figure 2B). The second region containing viral proteins was found to co-locate within viral fractions 14 to 17. Based on biochemical, viral and immunological properties, these infectious virus particles were defined as real viral particles or R-particles. R-particles have: (1) settled at 35–36% sucrose gradient and contained all four structural protein bands (VP1, 2, 3 and 4) observed in the SDS-PAGE as its unique biochemical property; (2) high infectivity observed in the TCID_50_ assay as their viral property; and (3) single VP2 protein band in the Western blot analysis (fractions 14 and 17 in Figure 2B). To identify differences between these two types of CVA16 viral particles, fractions 10–11 and 14–17 were pooled as P-particles and R-particles, respectively. These particle fractions were individually diafiltrated using PBS and then concentrated to 20 mL as virus particle solutions. P- and R-particle protein concentrations were found to be 18 and 40 µg/mL, respectively. In five batches of bioreactor runs, protein concentration of R-particles was consistently higher than that of P-particles with a ratio of 2∶1. This ratio is very different from those found in EV71 virus production that had 3∶7 ratios for R- and P-particles, respectively [27].

### Biophysical characterization of CVA16 viral particles by transmission electron microscopy (TEM)

**Figure 3:**
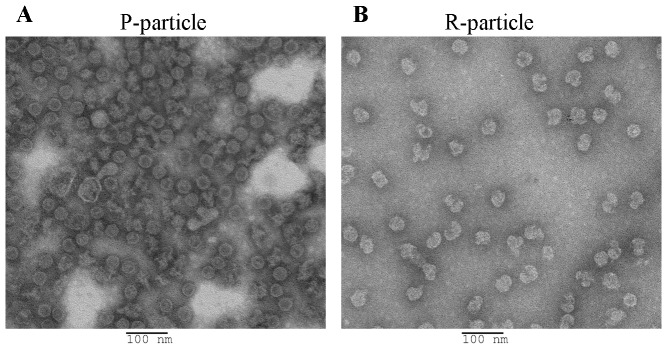
Transmission electron microscopy imaging of formalin-inactivated CVA16 viral particles. (**A**) Fraction 10 was empty and had a defective particle (P-particle) structure. (**B**) Fraction 16 was full and had a solid particle (R-particle) structure.

The physical structures of CVA16 P- and R-particles were revealed by TEM analysis. For biosafety reasons, purified virus solutions were individually inactivated by formalin solution (v/v 1∶4000 dilution) at 37°C for 3 days. After preparation as described in Materials and Methods, both samples were analyzed by TEM and found to have some irregular icosahedral particle structures (Fig. 3). Formalin-inactivated P-particles (Fig. 3A) and R-particles (Fig. 3B) were similar; the icosahedral structure of both particles was destroyed by formalin-inactivation. Both P- and R-particles were found to have diameters of approximately 30–32 nm, which are very similar to enteroviruses of the *Piconaviridae* family [30]–[35].

### The viral protein compositions of CVA16 viral particles

**Figure 4:**
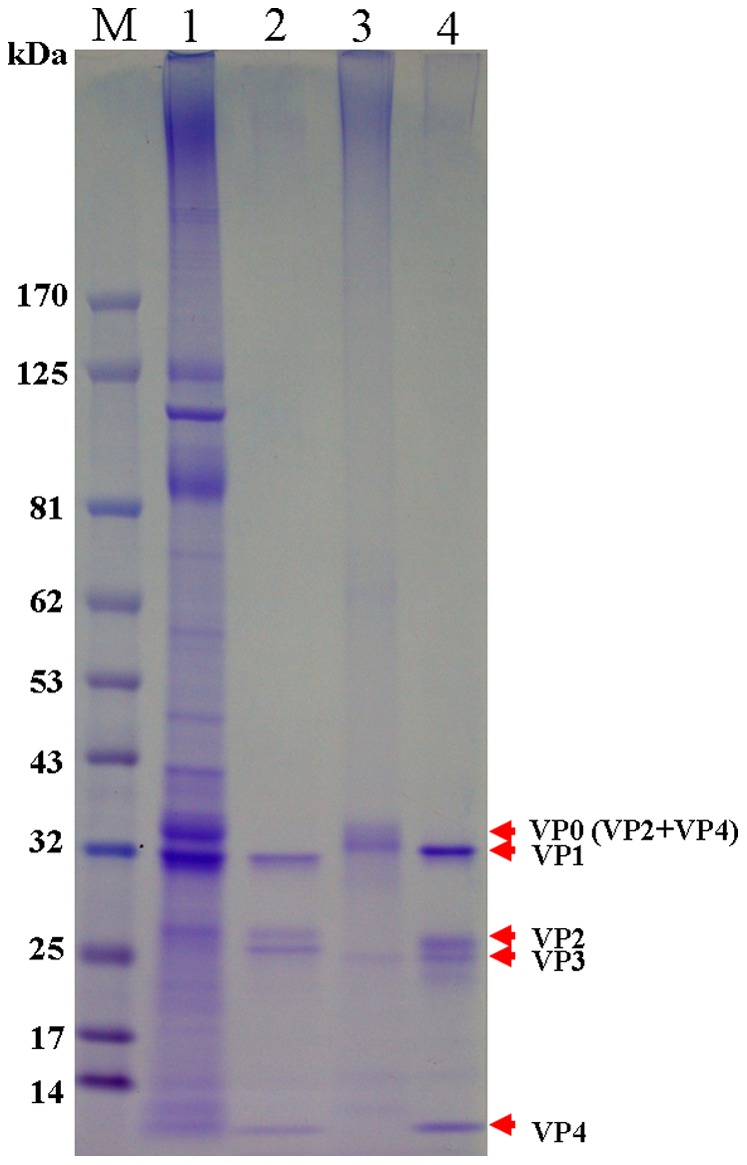
SDS-PAGE analysis of viral antigen composition of CVA16 and EV71 viral particles. CVA16 P-particles (lane 1), CVA16 R-particles (lane 2), EV71 P-particles (lane 3), and EV71 R-particles (lane 4) were analyzed on a NuPAGE 4–12% Birs-Tris Gel.

Both sucrose gradient zonal ultracentrifugation and TEM analysis demonstrated that there were two types of CVA16 viral particles produced by Vero cells grown in the serum-free microcarrier bioreactor system. Like the defective particles found in EV71 [27], CVA16 P-particles were shown to contain three major protein bands with molecular weights (MWs) of 36 kDa (VP0), 32 kDa (VP1) and 27 kDa (VP3) (Fig. 4, lane 1). Some high molecular weight proteins indicate that the P1 polypeptide was most likely incompletely processed during viral assembly and packaging. The R-particles viral proteins were separated and analyzed by SDS-PAGE, and found to contain four major protein bands with MWs similar to those found in EV71 infectious particles (Fig. 4, lanes 2 & 4). These four major protein bands correspond with human enterovirus capsid proteins VP1 (33 kDa), VP2 (28 kDa), VP3 (27 kDa) and VP4 (8 kDa) based on their predicted protein sequences (Fig. 4, lane 2). Western blot was performed using a commercially-available VP2-specific monoclonal antibody MAb979 to confirm the identity of the 36 kDa protein band to be incompletely-processed VP2−VP4 (VP0) (Fig. 4, lanes 1 & 3). These results suggest that P-particles are composed of incompletely-processed viral capsid proteins like those found in EV71 [27]. Taken together, these results indicate that the two viral particles have different protein compositions. Furthermore, the immature capsid constructed by incompletely-processed viral proteins can still form the particle structure.

### RT-PCR analysis of the CVA16 viral RNA content

**Figure 5:**
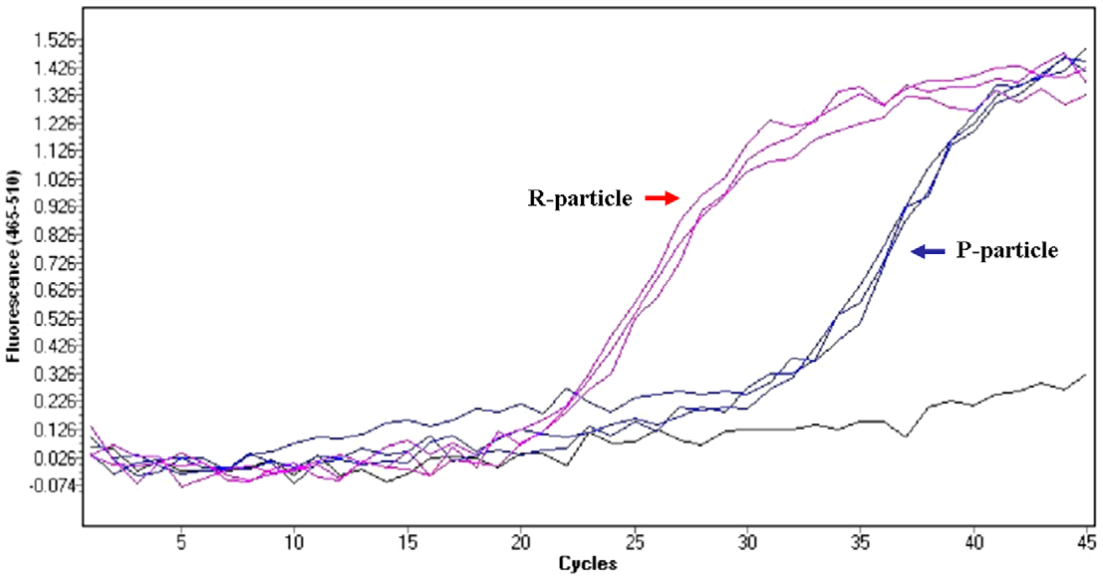
CVA16 viral RNA content measured by RT-PCR. The results of quantitative RT-PCR using primers specific for a 60 bp region of the CVA16 VP2 gene are reported for the P-particle and R-particle by the blue line and red line, respectively.

As described above, P-particles have low or no virus infectivity, contain incompletely-processed viral polypeptides, and are considerably lighter than R-particles based on sucrose gradient ultracentrifugation. Viral RNA was individually extracted from both CVA16 viral particles to confirm that the differences in physical structure were due to RNA contents and packaging. As described in the Materials and Methods, CVA16 RNA contents were measured by quantitative RT-PCR using specific primers to amplify a 60 bp region of the VP2 gene. Like the EV71 study [27], the signal for the R-particle VP1 RNA was detectable after 22 cycles. In contrast, the P-particle gave a detectable signal only after 32 cycles (Figure 5). The low P-particle RNA content is consistent with its low infectivity.

### Immunogenicity studies of CVA16 viral particles

It is of interest to investigate whether these two types of formalin-inactivated CVA16 viral particles could generate strong and efficacious immune responses. Five groups of mice were immunized with different amounts of inactivated particles formulated with Alum and boosted 2× with the same dosage every 2 weeks. Mouse anti-sera from the groups of mice immunized with either PBS or formalin-inactivated P-particles failed to induce CVA16 virus neutralization (Table 1). This was a surprise since formalin-inactivated EV71 P-particles were found to be capable of eliciting strong EV71-specific neutralizing antibody responses in both mouse and rabbit immunogenicity studies [27]. As shown in Table 1, the formalin-inactivated R-particle induced CVA16-specific neutralizing antibody responses with an average titer of 1/128. In the previous EV71 study [27], the EV71-specific neutralization titer of mouse anti-sera generated from formalin-inactivated EV71 R-particles was found to be around 1/2000. Based on the current results, formalin inactivation may destroy some neutralization epitopes of CVA16 that could cause poor virus neutralizing antibody responses. This is supported by our previous study [30] that the virus neutralization epitopes of some EV71 virus strain were very sensitive to chemical inactivation such as formalin, UV and heat-treatment. The current EV71 vaccine strain was selected because based on its conformational epitopes remained to be immunogenic after formalin-inactivation and had ability to elicit high virus neutralizing antibody responses in most animal immunogenicity studies.

**Table 1:** Enterovirus neutralization titers of a pool of 6 mouse anti-sera generated against either CVA16 or EV71 formalin-inactivated viral particles as measured by TCID_50_ neutralization assay.

Group	Anti-sera	Total protein (µg)	TCID_50_ neutralization titer	
			CVA16	EV71 (B4)
1	PBS	0	**<10**	**<10**
2	CVA16 P-particles + Alum	0.5	**<10**	**<10**
3	CVA16 P-particles + Alum	2.5	**<10**	**<10**
4	CVA16 R-particles + Alum	0.5	**64**	**<10**
5	CVA16 R-particles + Alum	2.5	**128**	**<10**
6	EV71 P-particles + Alum	2.5	**<10**	**1280**
7	EV71 R-particles + Alum	2.5	**<10**	**2560**

To investigate whether formalin-inactivated CVA16 is truly a poor immunogen or if the poor antibody responses are due to mouse immunogenicity problems, two groups of rabbits (3 rabbits per group) were immunized i.m. 3 times every 2 weeks with 2.5 µg of formalin-inactivated CVA16 particles formulated with alum. Anti-sera obtained from two rabbits immunized with formalin-inactivated R-particles were found to have 1/128 and 1/512 neutralization titers, and anti-sera from rabbits immunized with the same amount of formalin-inactivated P-particles formulated with alum were found to have either no or little neutralization titers (1/16) against CVA16 isolates (Table 2). These neutralization titers were far too low compared to those titers obtained from EV71 R-particles in previous studies [23], [27]. These titers were found to be approximately 1/2000 and 1/32,000 from mouse and rabbit immunogenicity studies, respectively.

**Table 2:** Enterovirus neutralization titers of rabbit anti-sera generated against either CVA16 or EV71 formalin-inactivated particles as measured by TCID_50_ neutralization assay.

Group	Sample	Total protein (µg)	TCID_50_ neutralization titer	
			CVA16	EV71 (B4)
1	PBS	0	**<10**	**<10**
2	CVA16 P-particles+Alum	2.5	**16**	**<10**
3	CVA16 R-particles+Alum	2.5	**256**	**<10**
4	EV71 P-particles+Alum	2.5	**<10**	**5120**
5	EV71 R-particles+Alum	2.5	**16**	**32000**

To investigate what factors caused these poor neutralizing antibody responses, we can rule out vaccine precipitation since no obvious viral particle aggregates were observed in the solution containing formalin-treated CVA16 virion. Both formalin-inactivated CVA16 particles formulated with alum are immunogenic since they can elicit relatively good IgG titer (1/6400) against CVA16 as determined by ELISA (data not shown). Therefore, it seems most likely that the formalin inactivation process destroys some critical virus neutralization epitopes. Other inactivation methods should be investigated.

It is of interest to determine whether these CVA16 virus neutralizing antibodies can cross-neutralize EV71. Mouse and rabbit sera were tested in the neutralization assay and found to be inactive against EV71 at 1/10 dilution (Tables 1 and 2). The current results indicate that neutralizing antibody responses to formalin-inactivated CVA16 vaccine candidates are virus-specific. Mao *et al.* [25] and our unpublished results also demonstrated that formalin-inactivated EV71 vaccine candidates induced EV71- specific neutralizing antibody responses that had no or poor cross-neutralization activity against CVA16. Taking these results together, a bi-valent EV71/CVA16 vaccine should be developed and tested against human enteroviruses causing HFMD.

### Linear immunodominant epitope mapping

**Figure 6:**
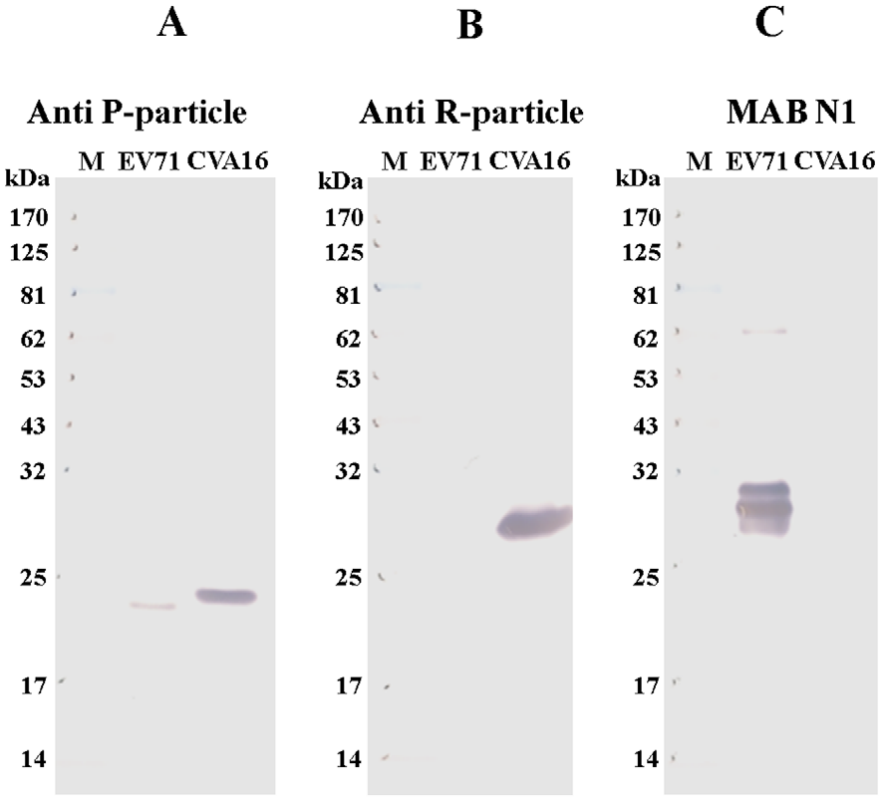
Western blot analysis of reactivity of enterovirus antigens with mouse anti-sera raised against formalin-inactivated CVA16 particles. R-particles derived from CVA16 and EV71 were separated on a NuPAGE 4–12% Bis-Tris Gel and analyzed using different antibodies: (**A**) anti-sera generated from formalin-inactivated CVA16 P-particles; (**B**) anti-sera generated from formalin-inactivated CVA16 R-particles; and (**C**) EV71-specific monoclonal antibody MAb N1.

Although current results indicate both mouse and rabbit anti-sera have CVA16 virus-specific neutralizing antibodies, it was worth investigating whether these antibodies could cross-react with EV71 viral proteins. As shown in Fig. 6A and B, Western blot analyses indicated that anti-sera generated from formalin-inactivated P-particles react with VP3 of both CVA16 (strongly) and EV71 (weakly). In contrast, anti-sera generated from formalin-inactivated R-particles reacted only with VP1 of CVA16 and did not recognize any viral proteins from EV71. Both mouse anti-sera and monoclonal antibody N1 generated from the EV71 vaccine candidate failed to recognize any structural proteins of CVA16 (Fig. 6C). These results strongly support that formalin-inactivated enterovirus elicit genotype virus-specific neutralizing antibody responses as described above.

**Figure 7:**
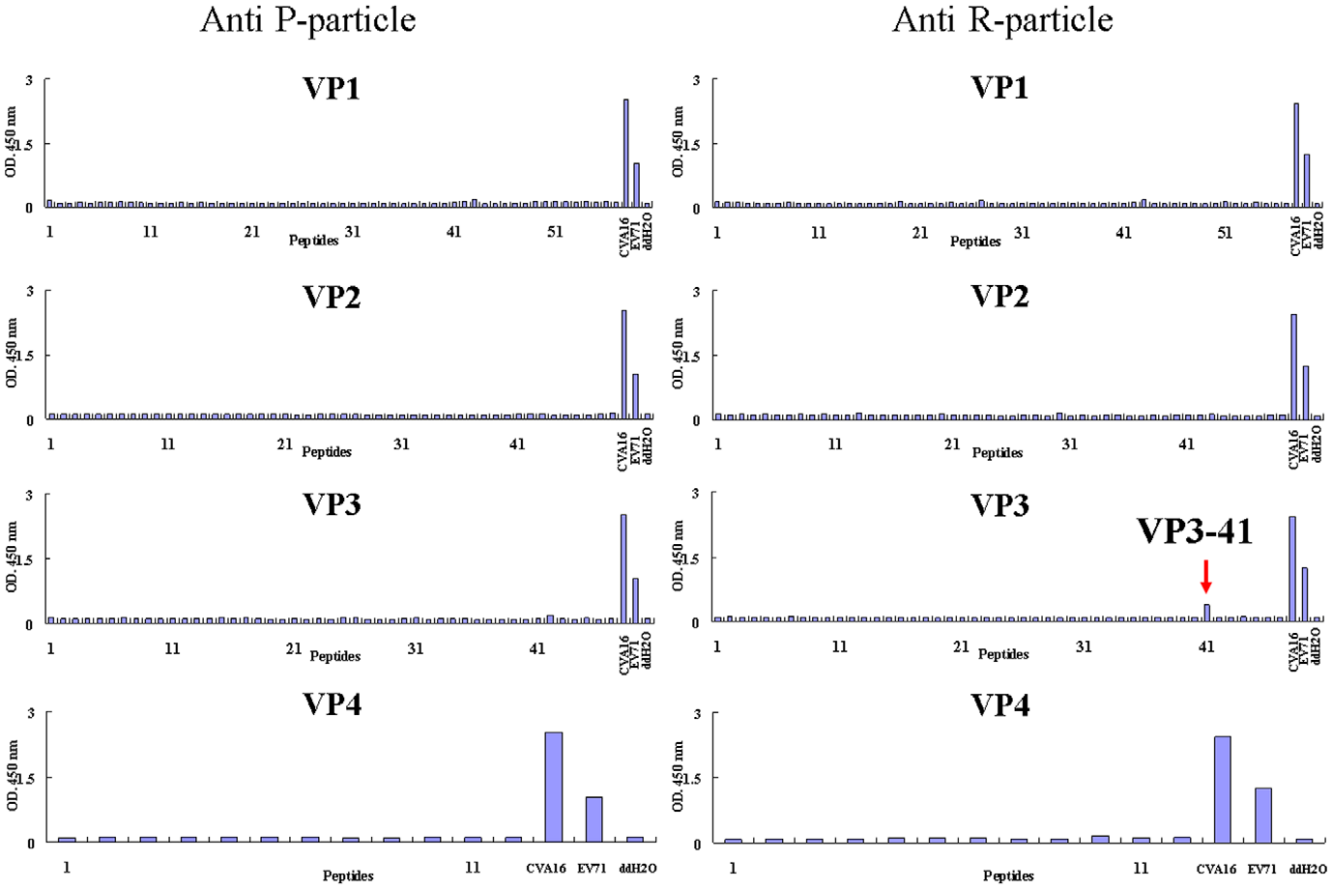
Immunodominant B-cell linear epitope mapping against mouse anti-sera raised against CVA16 formalin-inactivated P-particles (left panel) and formalin-inactivated R-particles (right panel). Peptide-ELISA format was used in covering all VP1, VP2, VP3 and VP4 sequences of CVA16.

Since anti-sera generated from formalin-inactivated CVA16 particles recognize different CVA16 viral proteins in Western blot analyses, it is of interest to investigate whether the two types of particles generated different spectrum of antibodies that recognize different linear immunodominant epitopes. The specificity of these anti-sera was screened by peptide-ELISA for their reactivity with 153 overlapping synthetic peptides (15-mer) that covered all 4 structural proteins (VP1−VP4). Rabbit anti-sera generated from either formalin-inactivated R- or P-particles failed to react with any synthetic peptide in peptide-ELISA studies, but reacted very well with formalin-inactivated EV71 and CVA16 (data not shown). As shown in Fig. 7, only mouse anti-sera raised against formalin-inactivated R-particles reacted weakly with the VP3-41 peptide corresponding to residues 176–190 (HYRAHARAGYFDYYT). These results are very different from mouse anti-EV71 sera that react strongly with the linear immunodominant neutralization epitope VP1-43 [27]. The different immune responses elicited, the current epitope mapping results and Western blot analyses all strongly suggest that the configuration and antigenic determinants in formalin-inactivated P- and R-particles of CVA16 are significantly different.

## Discussion

In Asia, human EV71 and CVA16 are most commonly identified as the viruses associated with HFMD outbreaks. Young children (under 5 years) infected with CVA16 develop fever and painful blisters in the mouth and on the hands and feet, but recover quickly within a week and without long-term problems in most cases. However, EV71 epidemics in Taiwan in 1998, 2001, 2005 and 2008 caused children to develop more severe symptoms such as neurological disease and death. For these reasons, we initiated Vero cell-based EV71 vaccine development in 2007 and have successfully produced a formalin-inactivated whole virion EV71 vaccine candidate currently being evaluated in human phase 1 clinical trials [23]. The preliminary results from current trials indicate that human sera generated from formalin-inactivated EV71 virions have low and no cross-neutralizing antibodies against CVA16. In addition, Mao *et al.* [25] and our unpublished results showed that animal anti-sera (mouse, rabbit and macaque) generated from formalin-inactivated EV71 had low or no cross-neutralization against CVA16. Therefore, it is of interest to investigate whether CVA16 vaccine candidates could elicit cross-neutralizing antibody responses against EV71.

In this study, we used a similar approach to develop a Vero cell-based formalin-inactivated whole virion CVA16 vaccine candidate. From 3 clinical CVA16 isolates we have identified and selected a potential vaccine strain (N5079) which has consistently shown faster growth and higher virus titer in serum-free medium. Fast-growth CVA16 clone (5079N/R-P2) has been tested for genetic stability in passage studies and has been found to be highly stable as shown by nucleotide sequence analysis. Although this vaccine strain can consistently provide virus titer >10^6^ TCID_50_ per mL, the bioprocess needs to be optimized since the current virus titer is lower than those found in the previous EV71 studies [23], [27].

Using 5 L bioreactor and microcarrier cell culture systems as the up-stream process and sucrose gradient zonal ultracentrifugation as the downstream purification, two types of particles were separated and purified: defective particles (P-particles) and infectious particles (R-particles). The biophysical and biochemical observations are very similar to EV71 and those of poliovirus studies [27], [31]–[36]. Two types of poliovirus structures (D- and C-antigens) were observed and characterized by electron microscopy and biochemical assays [32]–[34]. The crystal structures of EV71 particles were also recently solved [35]–[36]. Using RT-PCR analysis, the R-particle in this study similar to the D-antigen has a high viral RNA content and a full particle structure. In contrast, the P-particles found in both EV71 and CVA16 studies are similar to the C-antigen which has an empty particle structure and lacks of RNA content [32]–[36].

When we compared the total protein yield of these CVA16 viral particles from 5 batches of bioreactor runs, the ratio of P-particles to R-particles was consistently 1∶2. This ratio is different from those found in EV71 studies [23], [27] wherein the ratio was 7∶3. Currently we are investigating the factors that could influence this ratio and the final infectious virus yield. Biophysical and biochemical analyses were performed to further characterize these particles. Through EM imaging, the two formalin-inactivated CVA16 particles were found to have very similarly-sized icosahedral structures (Fig. 3). EM imaging also revealed some CVA16 particles containing irregular icosahedral structure (30–32 nm in diameter) that could be due to formalin inactivation. In contrast, EV71 particles were found to have different sizes ranging from 31–33 nm and 33–35 nm for P-particles and R-particles respectively [27], [35]–[36]. This size difference is due to differences in the composition of viral protein components and viral RNA contents. Generally, *Picornaviridae* virus morphogenesis begins with freshly-translated P1 polypeptide forming the pre-virion that requires specific cleavage of the P1 polypeptide into VP0, VP1 and VP3 proteins by the viral non-structural protein, 3CD protease. The final virion is assembled when VP0 protein is then cleaved into VP2 and VP4 by autocatalytic action that involves viral RNA [31], [37]. Based on biochemical (SDS-PAGE and Western blot) analyses, CVA16 P-particles were found to be immature particles in which the P1 polypeptide and VP0 protein (VP2+VP4) was incompletely processed (Fig. 4, lane 1). Quantitative RT-PCR studies of the VP1 gene content also suggest that little amounts of CVA16 viral RNA were packaged into these immature and defective P-particles. The current results indicate that this CVA16 vaccine strain produces a significant amount of defective (non-infectious) CVA16 particles similar to those found in the previous EV71 study (Fig. 4, lanes 1 & 3) [27].

Surprisingly, formalin-inactivated P-particles formulated with alum elicited no titer or very low titer (1/16) of virus neutralizing antibody responses against CVA16 in BALB/c mice and rabbits (Tables 1 and 2), respectively. In contrast, alum formulated formalin-inactivated CVA16 R-particles could induce neutralizing antibody responses in both rabbit and mice, but the neutralization titers were found to be low compared to those obtained with EV71 vaccine candidate (Tables 1 and 2). In addition, current results and previous EV71 studies ([24] and our unpublished results) indicate that these virus neutralizing antibodies are virus-specific and have little or no cross-neutralization activity against other genotypes of human enterovirus. These results suggest that some of the neutralization epitopes in CVA16 are very sensitive to chemical modification and can be destroyed by formalin inactivation. Formalin inactivation is effective by cross-linking primary amino groups in viral proteins with aldehyde and other nearby nitrogen atoms. The inability of formalin-inactivated CVA16 P-particles to induce neutralizing antibody responses in mouse immunogenicity studies is very similar to that of the poliovirus C-antigen; and suggests that the conformations and interactions of viral proteins within the pentamers of P- and R-particles are likely to be different. This difference in particle structure and the interactions between the viral proteins were not easily observed by TEM. Differences in conformation were also observed in non-infectious empty particles and infectious viral particles of type 3 poliovirus using antigen-specific antibodies [32], [38] and EV71 crystal structures [35]–[36]. The CVA16 P-particle configuration that may be similar to the poliovirus C-antigen, is different from that of the D-antigen. This configuration could in turn influence the display of critical antigenic sites [32], [38]. The main antigenic sites and neutralization epitopes of poliovirus have been identified as conformational and are located in the VP1, VP2 and VP3 regions [38]. Current results from virus neutralization assay and linear epitope mapping suggest that CVA16 virus neutralization epitopes are also most likely conformational since rabbit anti-sera did not react with any linear CVA16 peptides and only one mouse linear epitope was identified within residues 176–190 of VP3. These findings are also very different from EV71 virus studies. Mouse immunodominant linear neutralization epitopes of EV71 are identified as residues 208–222 and 240–260 of the VP1 capsid protein [20], [21], [39]–[41]. Mouse neutralizing antibodies induced by formalin-inactivated CVA16 R-particles recognized VP1 of CVA16 but not EV71 in Western blot analyses (Fig. 5B). Mouse anti-sera also failed to recognize these important epitopes of VP1 during epitope mapping (Fig. 7). There could be two reasons for the failure of linear epitope mapping: (1) the surface exposed fragments and/or peptides of VP1 are not immunogenic, so no B-cell recognize them for antibody production; (2) antibodies generated against formalin-modified epitopes would not react with peptides with native amino acids. Also it is not surprising since there are five and eight amino acid differences in neutralization epitopes between EV71 and CVA16 within VP1 residues 205–220 and 240–255 respectively [4], [42]–[43]. This observation is also supported by the fact that the epitope-specific (residue 205–220) monoclonal antibody N1 only recognizes VP1 of EV71 [39] and not CVA16 (Fig. 6C). All current results indicate that the configuration of formalin-inactivated CVA16 P-particles may be different enough to influence important antigenic and immunogenic sites for eliciting virus neutralizing antibody responses. In addition, EV71 formalin-inactivated P-particles could not elicit neutralizing antibody responses and would not be useful as CVA16 vaccine candidates in future.

In conclusion, the current findings and the full characterization of CVA16 viral particles provide valuable information for the development of cell-based formalin-inactivated CVA16 vaccine. Current mouse and rabbit immunological and serological data indicate that anti-sera raised against formalin-inactivated CVA16 vaccine candidates cannot cross-neutralize the EV71 virus, so a bi-valent EV71/CVA16 containing both viral particles should be considered for HFMD vaccine development. The proposed bi-valent EV71/CVA16 vaccine is also fully supported by the fact that EV71 immune responses can be recalled by exposure to CVA16 virus [44]–[45].

## References

[1] McMinn PC (2002) An overview of the evolution of enterovirus 71 and its clinical and public health significance. FEMS Microbiol Rev 26: 91–107. 12007645 10.1111/j.1574-6976.2002.tb00601.x

[2] Schmidt NJ, Lennette EH, Ho HH (1974) An apparently new enterovirus isolated from patients with disease of the central nervous system. J Infect Dis 129: 304–9. 4361245 10.1093/infdis/129.3.304

[3] Ho M, Chen ER, Hsu KH, Wu SJT, Chen KT, et al (1999) The Taiwan Enterovirus Epidemic Working Group. An epidemic of enterovirus 71 infection in Taiwan. N Engl J Med 341: 929–935. 10498487 10.1056/NEJM199909233411301

[4] Huang SW, Hsu YW, Smith DJ, Kiang D, Tsai HP, et al (2009) Reemergence of enterovirus 71 in 2008 in Taiwan: Dynamics of genetic and antigenic evolution from 1998 to 2008. J Clin Mincrobiol 47: 3653–3662. 10.1128/JCM.00630-09 PMC2772620 19776232

[5] Lin KH, Hwang KP, Ke GM, Wang CF, Ke LY, et al (2006) Evolution of EV71 genogroup in Taiwan from 1998 to 2005: an emerging of subgenogroup C4 of EV71. J Med Virol 78: 254–262. 16372302 10.1002/jmv.20534

[6] McMinn PC (2003) Enterovirus 71 in the Asia-Pacific region: An emerging cause of acute neurological disease in young children. Neurological journal of Southeast Asia 8: 57–63.

[7] Qiu J (2008) Enterovirus 71 infection: a new threat to global public health? Lancet Neurol 7: 868–869. 18848307 10.1016/S1474-4422(08)70207-2 PMC7128195

[8] Xu J, Qian Y, Wang S, Serrano JMG, Li W, et al (2010) EV71: An emerging infectious disease vaccine target in the Far East? Vaccine doi:10.1016/j.vaccine.2010.03.003. 10.1016/j.vaccine.2010.03.003 20304038

[9] Nishimura Y, Shimojima M, Tano Y, Miyamura T, Wakita T, et al (2009) Human P-selectin glycoprotein ligand-1 is a functional receptor for enterovirus 71. Nature Med 15: 794–797. 19543284 10.1038/nm.1961

[10] Yamayoshi S, Yamashita Y, Li J, Hanagata N, Minowa T, et al (2009) Scavenger receptor B2 is a cellular receptor for enterovirus 71. Nature Med 15: 798–801. 19543282 10.1038/nm.1992

[11] Lee MS, Chang LY (2010) Development of enterovirus 71 vaccines. Expert Rev Vaccines 9: 149–156. 20109026 10.1586/erv.09.152

[12] Arita M, Nagata N, Iwata N, Ami Y, Suzaki Y, et al (2007) An attenuated strain of enterovirus 71 belonging to genotype A showed a broad spectrum of antigenicity with attenuated neurovirulence in cynomolgus monkeys. J Virol 81: 9386–95. 17567701 10.1128/JVI.02856-06 PMC1951441

[13] Lin YC, Wu CN, Shih SR, Ho MS (2002) Characterization of a Vero cell-adapted virulent strain of enterovirus 71 suitable for use as a vaccine candidate. Vaccine 20: 2485–93. 12057603 10.1016/s0264-410x(02)00182-2

[14] Ong KC, Devi S, Cardosa MJ, Wong KT (2010) Formaldehyde-inactivated whole-virus vaccine protects a murine model of enterovirus 71 encephalomyetitis against disease. J Virol 84: 661–665. 19864378 10.1128/JVI.00999-09 PMC2798416

[15] Wu SC, Liu CC, Lian WC (2004) Optimization of microcarrier cell culture process for the inactivated enterovirus type 71 vaccine development. Vaccine 22: 3858–64. 15364432 10.1016/j.vaccine.2004.05.037

[16] Chung YC, Ho MS, Wu JC, Chen WJ, Huang JH, et al (2008) Immunization with virus-like particles of enterovirus 71 elicits potent immune responses and protects mice against lethal challenge. Vaccine 26: 1855–62. 18329759 10.1016/j.vaccine.2008.01.058

[17] Chiu CH, Chu C, He CC, Lin TY (2006) Protection of neonatal mice from lethal enterovirus 71 infection by maternal immunization with attenuated Salmonella enterica serovar Typhimurium expressing VP1 of enterovirus 71. Microbes Infect 8: 1671–8. 16815726 10.1016/j.micinf.2006.01.021

[18] Wu CN, Lin YC, Fann C, Liao NS, Shih SR, et al (2002) Protection against lethal enterovirus 71 infection in newborn mice by passive immunization with subunit VP1 vaccines and inactivated virus. Vaccine 20: 895–904. 10.1016/s0264-410x(01)00385-1 11738755

[19] Tung WS, Bakar SA, Sekawi Z, Rosli R (2007) DNA vaccine constructs against enterovirus 71 elicit immune response in mice. Genet Vaccines Ther 5: 6. 17445254 10.1186/1479-0556-5-6 PMC3225814

[20] Foo DG, Alonso S, Phoon MC, Ramachandran NP, Chow VT, Poh CL (2007) Identification of neutralizing linear epitopes from the VP1 capsid protein of Enterovirus 71 using synthetic peptides. Virus Res 125: 61–8. 17222936 10.1016/j.virusres.2006.12.005

[21] Guang DM, Foo W, Alonso S, Chow VTK, Poh CL (2007) Passive protection against lethal enterovirus 71 infection in newborn mice by neutralizing antibodies elicited by a synthetic peptide. Microes Infect 9: 1299–1306. 10.1016/j.micinf.2007.06.002 17890123

[22] Sivasamugham LA, Cardosa MJ, Tan WS, Yusoff K (2006) Recombinant Newcastle Disease virus capsids displaying enterovirus 71 VP1 fragment induce a strong immune response in rabbits. J Med Virol 78: 1096–104. 16789020 10.1002/jmv.20668

[23] Chou AH, Liu CC, Chang CP, Guo MS, Hsieh SY, et al (2012) Pilot scale production of highly efficacious and stable enterovirus 71 vaccine candidate. PLoS ONE 7 (4) e34834. 22529942 10.1371/journal.pone.0034834 PMC3328501

[24] Li YP, Liang ZL, Gao Q, Huang LR, Mao QY, et al (2012) Safety and immunogenicity of a novel human enterovirus 71 vaccine: a randomized, placebo-controlled, double-blind, phase 1 clinical trial. Vaccine doi:10.1016/j.vaccine.2012.03.010. 10.1016/j.vaccine.2012.03.010 22426327

[25] Mao Q, Li N, Yu X, Yao X, Li F, et al (2011) Antigenicity, animal protective effect and genetic characteristics of candidate vaccine strains of enterovirus 71. Arch Virol DOI 10.1007/s00705-011-1136-3. 10.1007/s00705-011-1136-3 21984267

[26] Liu CC, Lian WC, Butler M, Wu SC (2007) High immunogenic enterovirus 71 strain and its production using serum-free microcarrier Vero cell culture. Vaccine 25: 19–24. 16919374 10.1016/j.vaccine.2006.06.083

[27] Liu CC, Guo MS, Lin FHY, Hsiao KN, Chang KHW, et al (2011) Purification and characterization of enterovirus 71 viral particls produced from Vero cells grown in a serum-free microcarrier bioreactor system. PLoS ONE 6 (5) e20005. 21603631 10.1371/journal.pone.0020005 PMC3094384

[28] Reed LJ, Muench H (1938) A simple method of estimating 50 percent end-points. Am J Hyg 27: 493–497.

[29] Panezutti H, James O, Hansen EJ, Choi Y, Harkness RE, et al (1993) Identification of surface-exposed B-cell epitopes recognized by *H. Influenzae* type b P1-specific monoclonal antibody. Infect Immun 61: 1867–1872. 7682997 10.1128/iai.61.5.1867-1872.1993 PMC280777

[30] Chang JY, Chang CP, Tsai hh, Lee CD, Lian WC, et al (2012) Selection and characterization of vaccine strain for Enterovirus 71 vaccine devlopment. Vaccine 30: 703–711. 22142585 10.1016/j.vaccine.2011.11.087

[31] Curry S, Fry E, Blakemore W, Abu-Ghazaleh R, Jackson T, et al (1997) Dissecting the roles of VP0 cleavage and RNA packaging in picornavirus capsid stabilization: the structure of empty capsids of foot-and-mouth disease virus. J Virol 71: 9743–52. 9371640 10.1128/jvi.71.12.9743-9752.1997 PMC230284

[32] Ferguson M, Minor PD (1990) Differences in conformation of type 3 poliovirus antigenic sites on non-infectious empty particles and infectious virus. J Gen Virol 71: 1271–4. 1693661 10.1099/0022-1317-71-6-1271

[33] Hummeler K (1962) Identification of poliovirus particles of different antigenicity by specific agglutination as seen in the electron microscope. Virology 16: 84–90. 14449962 10.1016/0042-6822(62)90205-2

[34] Minor PD, Schild GC, Wood JM, Dandawate CN (1980) The preparation of specific immune sera against type 3 poliovirus D-antigen and C-antigen and the demonstration of two C-antigenic components in vaccine strain populations. J Gen Virol 51: 147–56. 6161996 10.1099/0022-1317-51-1-147

[35] Plevka P, Perera R, Cardosa J, Kuhn RJ, Rossmann MG. (2012) Crystal structure of human enterovirus 71. Scinecexpress www.sciencemag.org/cgi/content/full/science.1218713/DC1 Accessed 2012 March 1. 10.1126/science.1218713 PMC3448362 22383808

[36] Wang X, Peng W, Ren J, Hu Z, Xu J, et al (2012) A sensor-adaptor mechanism for enterovirus uncoating from structures of EV71. Nature Structure & Mol Biol doi:10.1038/nsmb.2255. 10.1038/nsmb.2255 PMC3378640 22388738

[37] Blom N, Hansen J, Blaas D, Brunak S (1996) Cleavage site analysis in picornaviral polyproteins: discovering cellular targets by neural networks. Protein Science 5: 2203–2216. 8931139 10.1002/pro.5560051107 PMC2143287

[38] Minor PD, Ferguson M, Katrak K, Wood D, John A, et al (1991) Antigenic structure of chimeras of type 1 and type 3 polioviruses involving antigenic sites 2, 3 and 4. J Gen Virol 72: 2475–81. 1717641 10.1099/0022-1317-72-10-2475

[39] Liu CC, Chou Ah, Lien SP, Lin HY, Liu SJ, et al (2011) Identification and characterization of a cross-neutralization epitope of EV71. Vaccine 29: 4362–73. 21501643 10.1016/j.vaccine.2011.04.010

[40] Chang HW, Liu CC, Lin MH, Ho HM, Yang YT, et al (2011) Generation of murine monoclonal antibodies which cross-neutralize human enterovirus 71 genotype B isolates. J Virol Methods 173: 189–95. 21315763 10.1016/j.jviromet.2011.02.003

[41] Chang GH, Luo YJ, Wu XY, Si BY, Lin L, et al (2010) Monoclonal antibody induced with inactived EV71-Hn2 virus protects mice against lethal EV71-Hn2 virus infection. Virol J 7: 106. 20500892 10.1186/1743-422X-7-106 PMC2887433

[42] Brown BA, Pallansch MA (1995) Complete nucleotide sequence of enterovirus 71 is distinct from poliovirus. Virus Res 39: 195–205. 8837884 10.1016/0168-1702(95)00087-9

[43] Tan EL, Chow VT, Quak SH, Yeo WC, Poh CL (2008) Development of multplex real-time hybridization probe reverse transcriptase polymerase chain reaction for specific detection and differentiation of EV71 and coxsackievirus A16. Diagn. Microbiol Infect Dis 61: 294–301. 10.1016/j.diagmicrobio.2008.02.009 18394844

[44] Wu TC, Wang YF, Lee YP, Wang JR, Liu CC, et al (2007) Immunity to avirulent enterovirus 71 and coxsackie A16 virus potects against enterovirus 71 infection in mice. J Virol 81: 10310–15. 17626076 10.1128/JVI.00372-07 PMC2045469

[45] Lin Y, Wen K, Pan Y, Wang Y, Che X, et al (2011) Cross-reactivity of anti-EV71 IgM and neutralizing antibody in series aera of patients infected with enterovirus 71 and coxsackievirus A16. J Immunoassay Immunochemistry 32: 233–43. 10.1080/15321819.2011.559297 21574094

